# The 12-Item Pruritus Severity Scale – Determining the Severity Bands

**DOI:** 10.3389/fmed.2020.614005

**Published:** 2020-12-17

**Authors:** Katarzyna Stępień, Adam Reich

**Affiliations:** Department of Dermatology, Institute of Medical Sciences, Medical College of Rzeszow University, Rzeszów, Poland

**Keywords:** itch, measurement, questionnaire, validation, pruritus

## Abstract

**Introduction:** Assessment of pruritus still remains a challenge due to its subjective character. Various itch questionnaires are widely used to evaluate the severity of pruritus. The aim of the current study was to define the cut off values for the 12-Item Pruritus Severity Scale (12-PSS).

**Methods:** A total of 240 patients (86 males and 154 females) in the age between 19 and 87 years (mean 52.9 ± 20.7 years) suffering from pruritic dermatological conditions were asked to assess their maximal pruritus with the 12-PSS, the Verbal Rating Scale (VRS) and the Numerical Rating Scale (NRS). All subjects also completed the Dermatology Life Quality Index (DLQI). VRS, NRS, and DLQI scorings were used as anchor measures to define cut-offs of 12-PSS.

**Results:** According to VRS, 43 (17.9%) patients suffered from mild, 96 (40%) from moderate, 65 (27.1%) from severe and 36 (15%) from very severe pruritus. Mean 12-PSS scoring for each VRS category was 7.6 ± 3.9, 10.4 ± 3.9, 13.0 ± 3.8, and 13.9 ± 3.8 points, respectively (*p* < 0.001). Each VRS category significantly differed from the others regarding the mean 12-PSS scoring, except the mean scoring of severe and very severe pruritus (*p* = 0.72). Thus, three pruritus severity categories of 12-PSS were defined with following score ranges: mild pruritus−3–6 points of 12-PSS, moderate pruritus−7–11 points of 12-PSS and severe pruritus−12–22 points of 12-PSS based on calculation of weighted κ coefficient against VRS, NRS, and DLQI as anchor measures.

**Conclusions:** The 12-PSS is able to differentiate between patients suffering from mild, moderate, and severe pruritus.

## Introduction

Pruritus is a subjective sensation which causes a desire to scratch. It is a common symptom of many dermatological as well as non-dermatological conditions. However, due to its subjective nature, its objective assessment, both in clinical trials as well as in routine daily practice, still remains a challenge (1). Among various methods of pruritus assessments, unidimensional scales, and itch questionnaires are most commonly used (1). The Special Interest Group (SIG) on itch questionnaires of the International Forum on the Study of Itch (IFSI) provided recommendations on the dimensions which should be addressed in an itch questionnaire in order to properly assess pruritus (2).

Recently, our group has developed the 12-Item Pruritus Severity Scale (12-PSS), which has been shown to be a valid and reliable assessment tool for patients suffering from dermatological itch (3). This questionnaire was validated in a group of 148 Polish patients with chronic pruritic dermatoses. It has also been successfully used by other researchers in patients with post-burn pruritus and uremic pruritus, indicating that the 12-PSS may also be applied in other pruritus types (4). As mentioned by Almeida et al. (5), the 12-PSS is probably not as specific as the 5-D itch scale for itch distribution, but it has the advantage of incorporating extent and consequences of scratching along with numerous aspects of quality of life. In a recent systematic review on itch questionnaires by Dominick et al., the 12-PSS was indicated as covering several dimensions of itch characteristics: localization, frequency, intensity, scratch response, affective qualities, sleep disturbances, and quality of life (6). In order to provide more data on the 12-PSS validity and give more information on interpretability of this scale we performed a study to define the cut off values for the 12-PSS.

## Materials and Methods

### Patients

Overall, 336 patients were asked to participate in this prospective study. Fifteen (4.5%) patients did not agree to participate, and further 81 (24.1%) individuals, after initial approval, were found not to have pruritus and subsequently were excluded from the study. Finally, a total of 240 subjects were included into this research projects. All active participants signed a written informed consent before any procedures related to the study.

The final analysis group consisted of 154 (64.2%) females and 86 (35.8%) males aged between 19 and 87 years (mean 50.6 ± 16.3 years). They suffered from psoriasis (*n* = 45, 18.7%), atopic dermatitis (AD) (*n* = 27, 11.2%), lichen planus (*n* = 61, 25.4%), cutaneous lupus erythematosus (CLE) (*n* = 79, 32.9%), eczema (*n* = 10, 4.2%), or other pruritic dermatoses (*n* = 18, 7.5%). All subjects suffered from pruritus for at least 6 week, i.e., they were all considered as having chronic pruritus of dermatological subtype.

### Study Design

All included patients were asked to assess their pruritus according to several methods in following order: the 5-point Verbal Rating Scale (VRS), the Numeric Rating Scale (NRS) (7, 8), and 12-PSS (3). The 12-PSS is a one-page instrument assessing different aspects of pruritus. The items are grouped into five domains: pruritus intensity, pruritus extent, frequency and duration of pruritus, impact of pruritus on daily activities and mood, and assessment of scratching (3). Patients evaluated their worst peak pruritus within 24 h before entering the study using VRS and NRS. Patients used the following descriptions within VRS: none, mild, moderate, severe, and very severe pruritus which were next translated into scores from 0 to 4. With NRS patients scored their pruritus from 0 (no pruritus) to 10 (worst imaginable pruritus), while with 12-PSS participants answered 12 questions referring to their pruritus and the total scoring ranged from 3 (the lowest pruritus intensity) to 22 points (the highest pruritus intensity). In addition, 202 patients completed the validated Polish version of the Dermatology Life Quality Index (DLQI) to assess their health-related quality of life (9). The scoring ranged from 0 (no impact of the disease on QoL) to 30 points (the worst impact of the disease on QoL). The study was accepted by Bioethics' Committee of Local Physician Chamber in Rzeszów, Poland.

### Statistical Analysis

All data were analyzed statistically using Statistica 12.0 (Statsoft, Kraków, Poland). Mean values, standard deviations, minimal, and maximal values, as well as frequencies, were calculated. Paired and unpaired Student's *t*-test, χ^2^ test with Yates correction, analysis of variance (ANOVA) with Scheffé's *post hoc* test and Spearman's rank correlation test were used where appropriate. The weighted κ coefficient of agreement was calculated for VRS, NRS, DLQI, and various sets of bands of 12-PSS total scores. The following assumptions regarding κ coefficient were made: <0 no agreement, 0–0.2—slight, 0.21–0.4—fair, 0.41–0.6—moderate, 0.61–0.8—substantial, and 0.81–1—almost perfect agreement. *P* < 0.05 were considered significant.

## Results

### Pruritus Intensity

**Figure 1:**
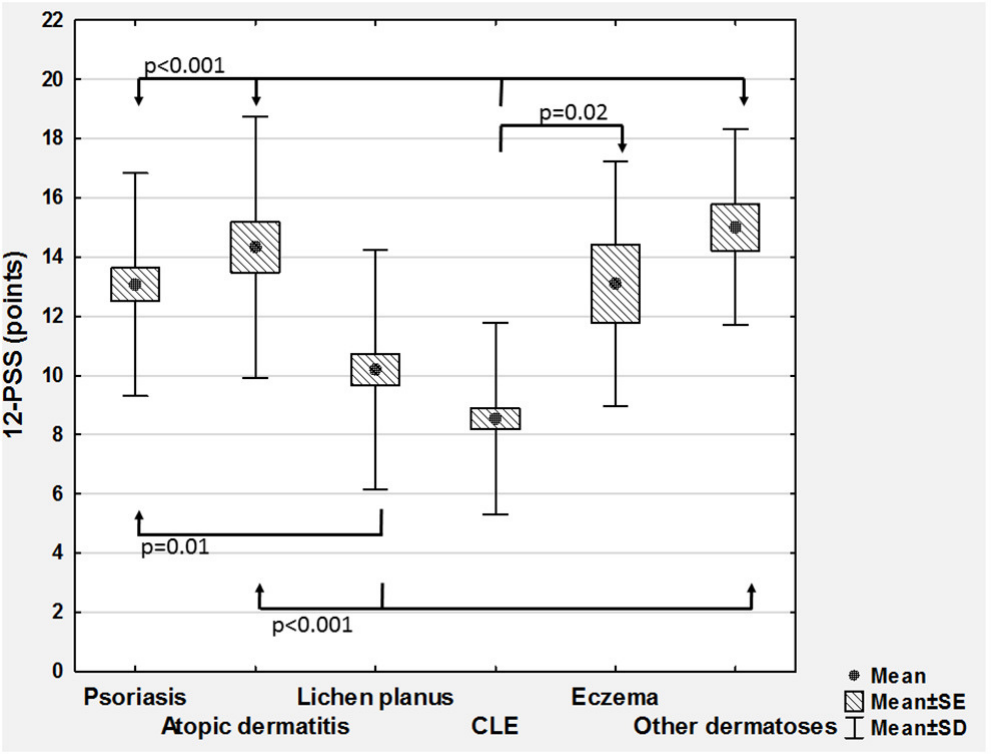
Comparison of 12-item Pruritus Severity Scale scoring (12-PSS) between various skin diseases (CLE, cutaneous lupus erythematosus; SD, standard deviation; SE, standard error).

According to VRS, 43 (17.9%) patients assessed their pruritus as mild, 96 (40%) as moderate, 65 (27.1%) as severe, and remaining 36 (15%) as very severe one. The mean pruritus intensity according to NRS was 5.9 ± 2.4 points (range 1–10 points) and according to 12-PSS: 11.1 ± 4.4 points (range 3–22 points; Table 1). All scales assessing pruritus intensity significantly correlated between themselves (VRS and NRS: ρ = 0.92, *p* < 0.001; VRS and 12-PSS: ρ = 0.49, *p* < 0.001; NRS and 12-PSS: ρ = 0.51, *p* < 0.001). There were no significant differences regarding pruritus intensity between males and females (Table 1), while considering age we observed weak, albeit significant correlation with 12-PSS indicating that older patients had slightly higher pruritus than younger individuals (ρ = 0.15, *p* = 0.02), however, such relationship was neither observed for VRS (ρ = 0.07, *p* = 0.28) nor for NRS (ρ = 0.07, *p* = 0.28). No significant differences were also observed between various skin diseases when itch intensity was assessed with NRS or VRS. However, using 12-PSS we found, that patients suffering from lichen planus or from CLE suffered from significantly less intense pruritus than patients with psoriasis (*p* = 0.01 and *p* < 0.0001, respectively) or AD (*p* = 0.002 and *p* < 0.0001, respectively; Figure 1). Patients with CLE experienced also significantly less severe pruritus than subjects with eczema (*p* = 0.04; Figure 1). Significant differences between various dermatoses regarding single questions of 12-PSS are demonstrated in Supplementary Table 1.

**Table 1:** Pruritus severity in studied patients.

	**VRS**	**NRS**	**12-PSS**	**DLQI**
All patients	2.4 ± 0.9 (1–4)	5.9 ± 2.4 (1–10)	11.1 ± 4.4 (0–30)	9.9 ± 7.3 (3–21)
Females	2.4 ± 1.0 (1–4)	6.0 ± 2.5 (1–10)	10.7 ± 4.4 (0–30)	9.3 ± 7.2 (3–20)
Males	2.3 ± 0.9 (1–4)	5.8 ± 2.3 (1–10)	11.8 ± 4.4 (0–28)	11.2 ± 7.5 (4–21)
p^*^	0.51	0.47	0.06	0.08
Psoriasis	2.3 ± 0.9 (1–4)	5.8 ± 2.3 (1–9)	13.1 ± 3.8 (3–28)	15.3 ± 7.7 (6–20)
AD	2.4 ± 1.0 (1–4)	6.0 ± 2.4 (1–10)	14.3 ± 4.4 (1–21)	11.8 ± 7.6 (5–21)
Lichen planus	2.7 ± 1.0 (1–4)	6.5 ± 2.7 (1–10)	10.2 ± 4.0 (0–22)	9.2 ± 5.9 (3–20)
CLE	2.2 ± 0.9 (1–4)	5.5 ± 2.3 (2–10)	8.5 ± 3.2 (0–30)	7.1 ± 7.0 (3–17)
Eczema	2.2 ± 1.0 (1–4)	5.3 ± 2.5 (1–9)	12.6 ± 5.1 (1–18)	11.0 ± 6.2 (7–19)
Other dermatoses	2.7 ± 0.8 (1–4)	6.3 ± 2.4 (1–10)	15.0 ± 3.3 (2–25)	13.9 ± 7.2 (5–20)
p^**^	0.05	0.21	<0.001	<0.001

AD, atopic dermatitis; CLE, cutaneous lupus erythematosus; DLQI, Dermatology Life Quality Index; NRS, Numeric Rating Scale; 12-PSS, 12-item Pruritus Severity Scale; VRS, Verbal Rating Scale;

* *p-values according to Student's T-test*,

** *p-values according to analysis of variance; min-max values provided in brackets*.

**Figure 2:**
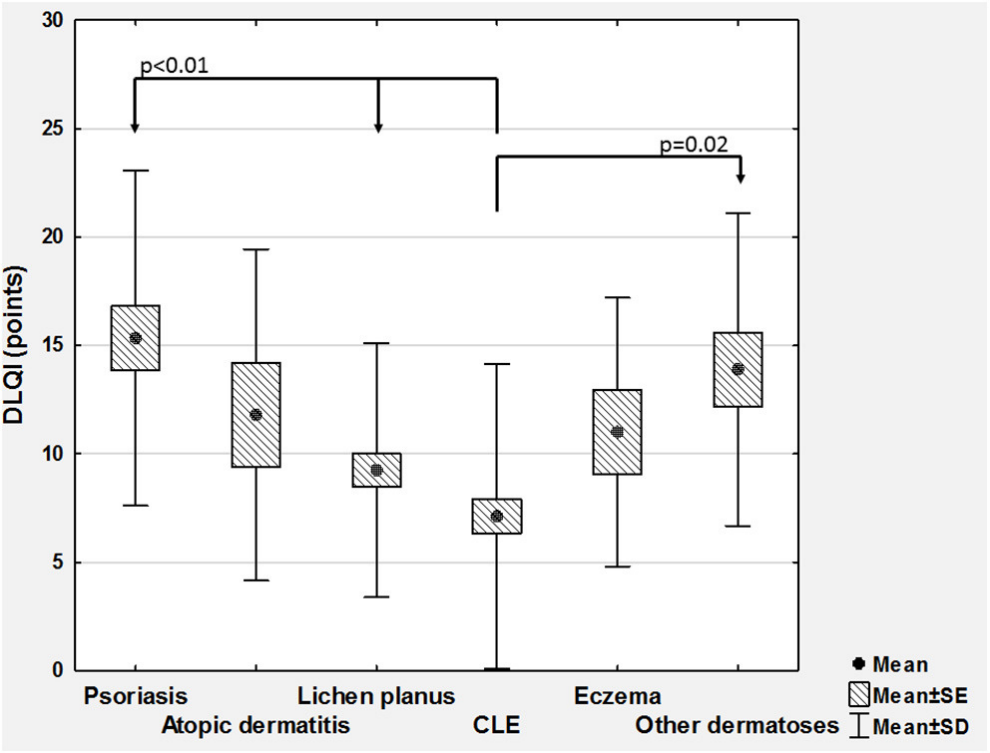
Comparison of Dermatology Life Quality Index scoring (DLQI) between various skin diseases (CLE, cutaneous lupus erythematosus; SD, standard deviation; SE, standard error).

Considering QoL, the mean DLQI scoring was 9.9 ± 7.3 points (range 0–30 points). Based on DLQI total scoring, 15 (7.4%) individuals reported no negative influence of skin disease on their QoL, 56 (27.7%) demonstrated small effect, 51 (25.2%) moderate effect, 58 (28.7%) very large, and remaining 22 (10.9%) extremely large effect on QoL. No significant differences regarding DLQI scoring was found between women and men (Table 1). However, similarly to 12-PSS, we were able to show significant differences between various skin conditions with DLQI (*p* < 0.001; Figure 2). DLQI total scoring also demonstrated much better correlation with 12-PSS scoring (ρ = 0.54) than with VRS (ρ = 0.24, *p* < 0.001) or NRS (ρ = 0.25, *p* < 0.001).

### Determination of Severity Bands

**Figure 3:**
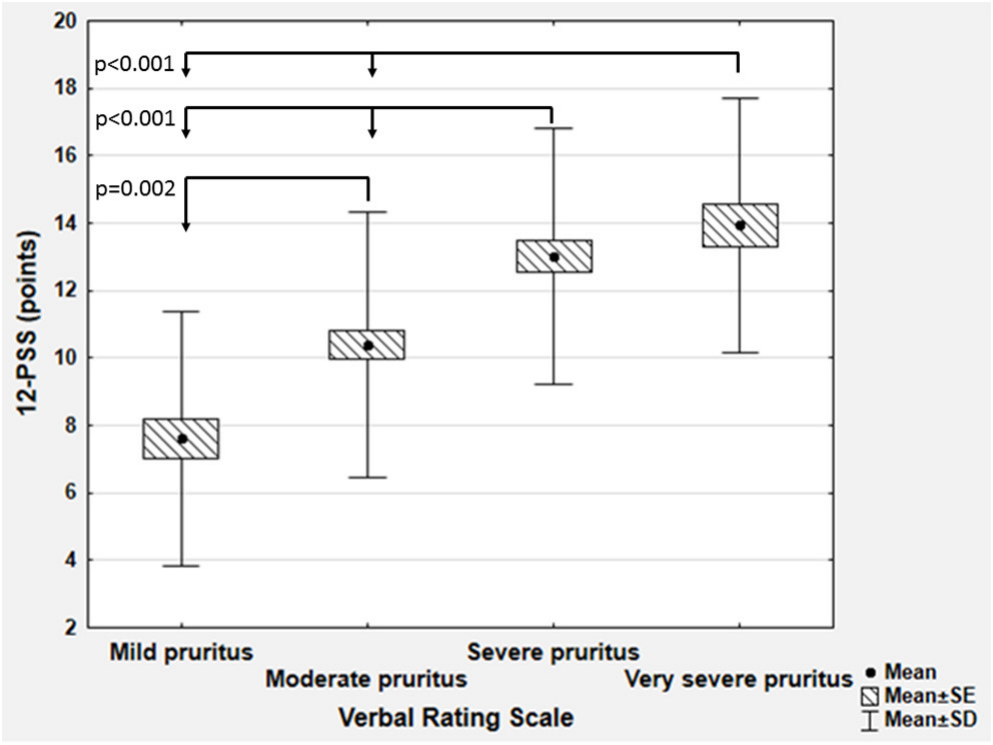
Comparison of 12-item Pruritus Severity Scale scoring (12-PSS) between various categories of the Verbal Rating Scale (SD, standard deviation; SE, standard error).

To determine the cut off values for 12-PSS we have used VRS categories as anchor measures. We observed that each VRS category significantly differed from the others regarding the mean 12-PSS scoring (*p* < 0.01 for each between group comparisons), except the mean total scoring in subjects who described their pruritus as severe or very severe (13.0 ± 3.8 vs. 13.9 ± 3.8, *p* = 0.72 according to Scheffe *post hoc* test; Figure 3). For that reason, only three pruritus severity categories of 12-PSS were further defined, namely mild, moderate, and severe pruritus. Based on mean and median 12-PSS scores for different VRS categories, several cut off values were proposed, which were next tested with weighted kappa coefficient. Calculation of weighted kappa coefficient for VRS revealed, that two sets of 12-PSS bands best define the categories of mild, moderate and severe pruritus, namely 3–6, 7–12, and 13–22 points (κ = 0.4889 ± 0.62, 95% CI 0.3674–0.6104), and 3–6, 7–11, and 12–22 points (κ = 0.4856 ± 0.622, 95%CI 0.3638–0.6074; Supplementary Table 2). To further elaborate, which set of bands should be chosen for future interpretation of 12-PSS scoring, we have also compared the predefined 12-PSS cut-offs with NRS (Supplementary Table 3) and DLQI (Supplementary Table 4) (10, 11). With both anchor measures following scoring: 3–6, 7–11, and 12–22 points defined better mild, moderate and severe pruritus (κ = 0.5339 ± 0.0673, 95%CI: 0.402–0.6658 for NRS, and κ = 0.4115 ± 0.0633, 95%CI: 0.2875–0.5355 for DLQI) than 3–6, 7–12, and 13–22 points (κ = 0.5188 ± 0.0675, 95%CI: 0.3865–0.6511 for NRS, and κ = 0.4098 ± 0.0641, 95%CI: 0.2842–0.5354 for DLQI) (Supplementary Tables 3, 4). The proposed set of bands (3–6, 7–11, and 12–22 points) also showed the highest correlation coefficients with VRS, NRS categories, and DLQI categories than any other tested grouping of 12-PSS scoring (Supplementary Table 5).

## Discussion

The 12-PSS consists of 12 questions, that assess different aspects of pruritus. The items are grouped into several domains related to pruritus: intensity, extent, frequency and duration of pruritus, impact of pruritus on daily activities, and mood and scratching assessment as a response to pruritus (3). As already suggested, 12-PSS is able to catch a more complex influence of pruritus on patient well-being than the Visual Analog Scale, VRS or NRS (6). Our previous study also demonstrated, that 12-PSS shows strong internal consistency, satisfactory convergent validity, as well as does not have a significant ceiling or bottom effect (3). Our current study also documented that 12-PSS is characterized by significant discriminative validity, as it enabled us to find marked differences regarding pruritus severity between various dermatoses. Remarkably, such differences could not be detected with VRS or NRS. Similarly, 12-PSS scoring demonstrated significant correlation with patients' age suggesting that older people may experience slightly more intense pruritus than younger individuals, a finding which could not be observed with VRS or NRS. Again, these findings may support the suggestion, that 12-PSS is able to assess broader aspects of the perception of pruritus intensity by particular patients (6, 12). In addition, results demonstrated by Samhan and Abdelhalim (4) indicated that 12-PSS shows also good responsiveness. These authors have used 12-PSS in patients with burn to assess the impact of low-energy extracorporeal shockwave therapy on pruritus and observed, that the post-treatment improvement of 12-PSS scoring was significantly greater in the study group than in the placebo group (scoring change of 5.9 points in treated patients vs. 1.8 points in control group, *p* < 0.001) (4).

In our current study we have focused on defining the grouping of 12-PSS scoring to provide more data on the interpretability of 12-PSS. Based on calculation of the weighted κ coefficient for different predefined sets of 12-PSS bands against various anchor parameters, we have proposed following 12-PSS scoring in relation to pruritus severity: mild pruritus−3–6 points, moderate pruritus−7–11 points, and severe pruritus—≥12 points. Such score grouping also showed the best correlations with VRS, NRS and DLQI categories. We do hope, that providing the cut offs value of 12-PSS, it will encourage other clinicians and researchers to use this scale more commonly, both in clinical practice as well as for scientific purposes.

However, we also have to mention some limitations of our results. First of all, we have defined the cut off values of 12-PSS only in patients with dermatological pruritus. It is unknown, if the same grouping can be employed for other pruritus subtypes or maybe the scoring should be interpreted in different way. Thus, physicians should use proposed scoring with caution when assessing patients with pruritus subtype other than a dermatological one, and further studies are needed to confirm that proposed set of bands is valid for patients with other pruritus types. As in our study we have analyzed only dermatological patients with pruritus concomitant to skin disorders, we have decided to evaluate QoL impairment with DLQI because this scale is widely used giving the possibility to compare our results with other authors, consists of only 10 questions, and the QoL impairment categories are well-defined. However, when assessing other pruritus subtypes, ItchyQol, an itch-specific QoL measure, would probably be more suitable (13). Nevertheless, it was shown, that DLQI scoring is strongly correlated with ItchyQol scoring (14), suggesting that at least in dermatological condition both scales may be used interchangeably and the use of DLQI in group of patients should not be considered as detrimental. Another aspects is the application of our results to other patient populations and to other languages. To date the questionnaire is available in following languages: English, Turkish, Persian, and Kannada. However, answers to questionnaire items may vary according to languages and current cut-offs, which were defined only using Polish version of 12-PSS, should be applied to another language versions with a great caution. Finally, we have to underline, that we assessed pruritus only once. It would be quite interesting to check, if the evaluation and interpretation of pruritus with 12-PSS changes over time with repeated assessments.

Despite the above mentioned limitations, we do believe that our results are convincing and provide a valid grouping of 12-PSS scoring. We hope, that giving a clear indication, how to interpret the results achieved with 12-PSS we will encourage other physicians and researchers to use this scale more commonly in their routine clinical and scientific work. Nevertheless, further studies are needed to confirm our results in various patient groups.

## Data Availability Statement

The original contributions presented in the study are included in the article/Supplementary Materials, further inquiries can be directed to the corresponding author.

## Ethics Statement

The studies involving human participants were reviewed and approved by Komisja Bioetyczna at Okręgowej Izbie Lekarskiej in Rzeszów. The patients/participants provided their written informed consent to participate in this study.

## Author Contributions

All authors listed have made a substantial, direct and intellectual contribution to the work, and approved it for publication.

## Conflict of Interest

The authors declare that the research was conducted in the absence of any commercial or financial relationships that could be construed as a potential conflict of interest. The reviewer CZ declared a past co-authorship with one of the authors AR to the handling Editor.
