# Supplementary material for: The 12-Item Pruritus Severity Scale – Determining the Severity Bands
Source: Front Med (Lausanne). 2020 Dec 17;7:614005. doi: 10.3389/fmed.2020.614005 (PMC7773774; doi:10.3389/fmed.2020.614005)
Supplement: Supplementary file 1 [file Table_1.docx]

**Supplementary table 1**

Significant differences between various dermatoses regarding single items of the 12-item Pruritus Severity Scale (data presented as means±standard deviations, analysis based Scheffé post hoc test)

|  | **Question** | **Significant differences between subgroups** |
| --- | --- | --- |
| 1. | How often did you feel pruritus within the last 3 days? (scoring range: 0 – 3 points) | Other dermatoses (2.4±0.7 points) vs. lichen planus (1.6±0.8 points, p=0.01), and vs. CLE (1.5±0.7 points, p<0.001) |
| 2. | Did pruritus hinder your ability to do simply things, like watching TV, hearing music, etc.? (scoring range: 0 – 1 point) | Other dermatoses (0.8±0.4 points) vs. lichen planus (0.3±0.5 points, p=0.04), and vs. CLE (0.2±0.4 points, p<0.001)  Psoriasis (0.6±0.5 points) vs. CLE (0.2±0.4 points, p<0.001) |
| 3. | Did you feel irritated or nervous because of your itching? (scoring range: 0 – 1 point) | CLE (0.6±0.5 points) vs. atopic dermatitis (0.9±0.3 points, p=0.04) |
| 4. | Did your pruritus cause you depressed? (scoring range: 0 – 1 point) | CLE (0.3±0.5 points) vs. psoriasis (0.7±0.5 points, p=0.002), and vs. other dermatoses (0.7±0.5 points, p=0.04) |
| 5. | Did your pruritus impede your work or learning abilities? (scoring range: 0 – 1 point) | CLE (0.3±0.6 points) vs. psoriasis (0.7±0.5 points, p=0.002), and vs. atopic dermatitis (0.8±0.4 points, p<0.001)  Lichen planus (0.4±0.5 points) vs. atopic dermatitis (0.8±0.4 points, p=0.01) |
| 6. | Did you scratch your skin because of itching? (scoring range: 0 – 1 point) | CLE (0.7±0.4 points) vs. psoriasis (1.0±0.0 point, p=0.003) |
| 7. | Did scratching bring you relief? (scoring range: 0 – 1 point) | No significant differences observed |
| 8. | Were you able to refrain from scratching? (scoring range: 0 – 1 point) | No significant differences observed |
| 9. | Did you wake up during last night because of pruritus? (scoring range: 0 – 3 points) | CLE (0.2±0.6 points) vs. psoriasis (0.9±1.0 points, p=0.003), vs. atopic dermatitis (1.3±1.1 points, p<0.001), vs. eczema (1.2±0.8 points, p=0.04), and vs. other dermatoses (1.4±1.0 points, p<0.001) |
| 10. | Could you assess the severity of your pruritus within last 3 days? (scoring range: 0 – 5 points) | CLE (2.6±1.0 points) vs. atopic dermatitis (3.5±1.2 points, p<0.01) |
| 11 | Could you indicate pruritus location? (scoring range: 0 – 3 points) | CLE (1.3±0.5 points) vs. psoriasis (2.0±0.7 points, p<0.001), vs. atopic dermatitis (2.1±0.8 points, p<0.001), vs. lichen planus (1.8±0.6 points, p<0.01), and vs. other dermatoses (2.2±0.7 points, p<0.001) |
| 12. | Are excorations or other scratch lesions present? (scoring range: 0 – 1 point) | CLE (0.2±0.4 points) vs. psoriasis (0.6±0.5 points, p<0.001), vs. atopic dermatitis (0.7±0.5 points, p<0.001), and vs. eczema (0.7±0.5 points, p=0.03) |
