# Supplementary material for: The 12-Item Pruritus Severity Scale – Determining the Severity Bands
Source: Front Med (Lausanne). 2020 Dec 17;7:614005. doi: 10.3389/fmed.2020.614005 (PMC7773774; doi:10.3389/fmed.2020.614005)
Supplement: Supplementary file 2 [file Table_2.docx]

**Supplementary table 2**

Calculated weighted kappa coefficients for different cut offs of the 12-item Pruritus Severity Scale against the Verbal Rating Scale as anchor measure.

| **Range** | | | **Observed Kappa** | **Standard error** | **Lower limit 95%** | **Upper limit 95%** |
| --- | --- | --- | --- | --- | --- | --- |
| Mild | Moderate | Severe |  |  |  |  |
| 3-6 | 7-11 | 12-22 | 0.49 | 0.06 | 0.36 | 0.61 |
| 3-6 | 7-12 | 13-22 | 0.49 | 0.06 | 0.37 | 0.61 |
| 3-6 | 7-13 | 14-22 | 0.48 | 0.06 | 0.36 | 0.6 |
| 3-7 | 8-10 | 11-22 | 0.46 | 0.06 | 0.35 | 0.58 |
| 3-7 | 8-11 | 12-22 | 0.48 | 0.06 | 0.36 | 0.59 |
| 3-7 | 8-12 | 13-22 | 0.48 | 0.06 | 0.36 | 0.59 |
| 3-7 | 8-13 | 14-22 | 0.47 | 0.06 | 0.35 | 0.58 |
| 3-8 | 9-10 | 11-22 | 0.44 | 0.05 | 0.34 | 0.55 |
| 3-8 | 9-11 | 12-22 | 0.45 | 0.05 | 0.34 | 0.56 |
| 3-8 | 9-12 | 13-22 | 0.45 | 0.05 | 0.35 | 0.56 |
| 3-8 | 9-13 | 14-22 | 0.44 | 0.05 | 0.34 | 0.55 |
| 3-9 | 10 | 11-22 | 0.38 | 0.05 | 0.28 | 0.47 |
| 3-9 | 10-11 | 12-22 | 0.38 | 0.05 | 0.29 | 0.48 |
| 3-9 | 10-12 | 13-22 | 0.39 | 0.05 | 0.29 | 0.48 |
| 3-9 | 10-13 | 14-22 | 0.37 | 0.05 | 0.28 | 0.47 |
