# Supplementary material for: The 12-Item Pruritus Severity Scale – Determining the Severity Bands
Source: Front Med (Lausanne). 2020 Dec 17;7:614005. doi: 10.3389/fmed.2020.614005 (PMC7773774; doi:10.3389/fmed.2020.614005)
Supplement: Supplementary file 3 [file Table_3.docx]

**Supplementary table 3**

Calculated weighted kappa coefficients for different cut offs of the 12-item Pruritus Severity Scale against the Numerical Rating Scale (divided as follows: mild pruritus: >0 and ≤3 points, moderate pruritus: >3 and <7 points, severe pruritus: ≥7 points [10]) as anchor measure.

| **Range** | | | **Observed Kappa** | **Standard error** | **Lower limit 95%** | **Upper limit 95%** |
| --- | --- | --- | --- | --- | --- | --- |
| Mild | Moderate | Severe |  |  |  |  |
| 3-6 | 7-11 | 12-22 | 0.53 | 0.07 | 0.4 | 0.67 |
| 3-6 | 7-12 | 13-22 | 0.52 | 0.07 | 0.39 | 0.65 |
| 3-6 | 7-13 | 14-22 | 0.5 | 0.07 | 0.37 | 0.63 |
| 3-7 | 8-10 | 11-22 | 0.51 | 0.06 | 0.39 | 0.64 |
| 3-7 | 8-11 | 12-22 | 0.51 | 0.06 | 0.39 | 0.63 |
| 3-7 | 8-12 | 13-22 | 0.5 | 0.06 | 0.37 | 0.62 |
| 3-7 | 8-13 | 14-22 | 0.48 | 0.06 | 0.36 | 0.6 |
| 3-8 | 9-10 | 11-22 | 0.49 | 0.06 | 0.38 | 0.6 |
| 3-8 | 9-11 | 12-22 | 0.47 | 0.06 | 0.37 | 0.6 |
| 3-8 | 9-12 | 13-22 | 0.47 | 0.06 | 0.36 | 0.58 |
| 3-8 | 9-13 | 14-22 | 0.46 | 0.06 | 0.35 | 0.57 |
| 3-9 | 10 | 11-22 | 0.41 | 0.05 | 0.31 | 0.52 |
| 3-9 | 10-11 | 12-22 | 0.41 | 0.05 | 0.3 | 0.51 |
| 3-9 | 10-12 | 13-22 | 0.4 | 0.05 | 0.29 | 0.5 |
| 3-9 | 10-13 | 14-22 | 0.38 | 0.05 | 0.28 | 0.48 |
