# Supplementary material for: The 12-Item Pruritus Severity Scale – Determining the Severity Bands
Source: Front Med (Lausanne). 2020 Dec 17;7:614005. doi: 10.3389/fmed.2020.614005 (PMC7773774; doi:10.3389/fmed.2020.614005)
Supplement: Supplementary file 4 [file Table_4.docx]

**Supplementary table 4**

Calculated weighted kappa coefficients for different cut offs of the 12-item Pruritus Severity Scale against the Dermatology Life Quality Index (divided as follows: small effect on patient’s life: 2-5 points, moderate effect on patient’s life: 6-10 points, very large or extremely large effect on patient’s life ≥ 11 points [11]) as anchor measure.

| **Range** | | | **Observed Kappa** | **Standard error** | **Lower limit 95%** | **Upper limit 95%** |
| --- | --- | --- | --- | --- | --- | --- |
| Mild | Moderate | Severe |  |  |  |  |
| 3-6 | 7-11 | 12-22 | 0.41 | 0.06 | 0.29 | 0.54 |
| 3-6 | 7-12 | 13-22 | 0.41 | 0.06 | 0.28 | 0.54 |
| 3-6 | 7-13 | 14-22 | 0.37 | 0.06 | 0.25 | 0.49 |
| 3-7 | 8-10 | 11-22 | 0.42 | 0.06 | 0.26 | 0.54 |
| 3-7 | 8-11 | 12-22 | 0.42 | 0.06 | 0.29 | 0.54 |
| 3-7 | 8-12 | 13-22 | 0.42 | 0.06 | 0.29 | 0.54 |
| 3-7 | 8-13 | 14-22 | 0.38 | 0.06 | 0.25 | 0.5 |
| 3-8 | 9-10 | 11-22 | 0.4 | 0.06 | 0.28 | 0.52 |
| 3-8 | 9-11 | 12-22 | 0.4 | 0.06 | 0.27 | 0.52 |
| 3-8 | 9-12 | 13-22 | 0.39 | 0.06 | 0.27 | 0.52 |
| 3-8 | 9-13 | 14-22 | 0.36 | 0.06 | 0.24 | 0.48 |
| 3-9 | 10 | 11-22 | 0.41 | 0.06 | 0.29 | 0.52 |
| 3-9 | 10-11 | 12-22 | 0.4 | 0.06 | 0.29 | 0.52 |
| 3-9 | 10-12 | 13-22 | 0.4 | 0.06 | 0.29 | 0.51 |
| 3-9 | 10-13 | 14-22 | 0.37 | 0.06 | 0.26 | 0.48 |
