# Supplementary material for: The 12-Item Pruritus Severity Scale – Determining the Severity Bands
Source: Front Med (Lausanne). 2020 Dec 17;7:614005. doi: 10.3389/fmed.2020.614005 (PMC7773774; doi:10.3389/fmed.2020.614005)
Supplement: Supplementary file 5 [file Table_5.docx]

**Supplementary table 5**

Correlation coefficients between various bands of 12-item Pruritus Severity Scale (12-PSS) scoring and other measures used to assess pruritus intensity (DLQI – Dermatology Life Quality Index, NRS – Numeric Rating Scale, VRS – Verbal Rating Scale).

| **Range of 12-PSS scoring** | | | **VRS** | **NRS** | **DLQI** | **DLQI - categories** |
| --- | --- | --- | --- | --- | --- | --- |
| Mild | Moderate | Severe |  |  |  |  |
| 3-6 | 7-11 | 12-22 | 0.46 | 0.49 | 0.53 | 0.51 |
| 3-6 | 7-12 | 13-22 | 0.46 | 0.48 | 0.52 | 0.5 |
| 3-6 | 7-13 | 14-22 | 0.46 | 0.48 | 0.46 | 0.45 |
| 3-7 | 8-10 | 11-22 | 0.45 | 0.48 | 0.53 | 0.5 |
| 3-7 | 8-11 | 12-22 | 0.46 | 0.48 | 0.52 | 0.49 |
| 3-7 | 8-12 | 13-22 | 0.46 | 0.47 | 0.52 | 0.49 |
| 3-7 | 8-13 | 14-22 | 0.46 | 0.48 | 0.47 | 0.45 |
| 3-8 | 9-10 | 11-22 | 0.45 | 0.5 | 0.52 | 0.48 |
| 3-8 | 9-11 | 12-22 | 0.46 | 0.5 | 0.52 | 0.48 |
| 3-8 | 9-12 | 13-22 | 0.46 | 0.49 | 0.51 | 0.48 |
| 3-8 | 9-13 | 14-22 | 0.46 | 0.5 | 0.47 | 0.44 |
| 3-9 | 10 | 11-22 | 0.4 | 0.44 | 0.51 | 0.48 |
| 3-9 | 10-11 | 12-22 | 0.41 | 0.44 | 0.5 | 0.47 |
| 3-9 | 10-12 | 13-22 | 0.41 | 0.44 | 0.5 | 0.47 |
| 3-9 | 10-13 | 14-22 | 0.41 | 0.45 | 0.48 | 0.45 |
